# A Novel Nonlinear Parameter Estimation Method of Soft Tissues

**DOI:** 10.1016/j.gpb.2017.09.003

**Published:** 2017-12-13

**Authors:** Qianqian Tong, Zhiyong Yuan, Mianlun Zheng, Xiangyun Liao, Weixu Zhu, Guian Zhang

**Affiliations:** 1 School of Computer, Wuhan University, Wuhan 430072, China; 2 Shenzhen Key Laboratory of Virtual Reality and Human Interaction Technology, Shenzhen Institutes of Advanced Technology, Chinese Academy of Sciences, Shenzhen 518000, China

**Keywords:** Nonlinear parameter estimation, Finite element method, Substitution parameters, Force correction, Self-adapting Levenberg–Marquardt algorithm

## Abstract

The elastic parameters of soft tissues are important for medical diagnosis and virtual surgery simulation. In this study, we propose a novel **nonlinear parameter estimation** method for soft tissues. Firstly, an in-house data acquisition platform was used to obtain external forces and their corresponding deformation values. To provide highly precise data for estimating nonlinear parameters, the measured forces were corrected using the constructed weighted combination forecasting model based on a support vector machine (WCFM_SVM). Secondly, a tetrahedral finite element parameter estimation model was established to describe the physical characteristics of soft tissues, using the **substitution parameters** of Young’s modulus and Poisson’s ratio to avoid solving complicated nonlinear problems. To improve the robustness of our model and avoid poor local minima, the initial parameters solved by a linear finite element model were introduced into the parameter estimation model. Finally, a **self-adapting Levenberg–Marquardt (LM) algorithm** was presented, which is capable of adaptively adjusting iterative parameters to solve the established parameter estimation model. The maximum absolute error of our WCFM_SVM model was less than 0.03 Newton, resulting in more accurate forces in comparison with other correction models tested. The maximum absolute error between the calculated and measured nodal displacements was less than 1.5 mm, demonstrating that our nonlinear parameters are precise.

## Introduction

Local changes in the mechanical properties of soft tissues may indicate the presence of tumor or other diseases [1], which could be detected by physicians using palpation. However, greatly depending on physicians’ experience, palpation is a subjective method for determining tissue properties [2]. Estimation of the elastic parameters of soft tissues plays a significant role in objective diagnosis of tumor or other diseases since elasticity is an important feature of soft tissues. Additionally, with the continued improvement of graphic calculation performance and virtual reality technology, virtual surgery [3], [4], [5] has become a hot topic. A precise model of soft tissues is essential to achieve immersive virtual surgery. Nevertheless, it remains challenging to build a precise model of soft tissues because soft tissues tend to possess rather complicated elastic behavior [6], [7].

To tackle the above issues, accurate estimation of soft tissue parameters is required. Young’s modulus and Poisson’s ratio are two important parameters describing the physical properties of materials. However, it is difficult and time-consuming to estimate these parameters, especially for nonlinear materials [8]. Many studies had been reported in modeling complex elasticity properties, and biomechanical models are widely used due to the high accuracy achieved [9], [10]. However, these models are complicated [11], [12], and the accuracy obtained relies on the accuracy of tissue geometry modeling and measurements of forces and deformations, as well as the rich excitation of material regimes [8]. Another commonly used model is the mass-spring model. Bao et al. [13] designed a virtual spring for every particle to obtain more realistic simulation results, and Takács et al. [14] utilized curve fitting methods to estimate mechanical parameters, achieving a good estimation of reaction force within the range of 0–4 mm, if the deformation shape function is appropriately approximated. Although it is easily fulfilled with no need for continuous parameterization and also satisfies real-time requirements, the mass-spring model exhibits poor fidelity. It is of note that minimally invasive parameter estimation tests had been reported on animals and humans based on data-driven approaches [15]. Given the changing material properties over time [16], images are used in recent studies to estimate the material properties of soft bodies. For instance, Mojsejenko et al. [17] estimated passive mechanical properties in a myocardial infarction from magnetic resonance imaging (MRI). In addition, Yang et al. [18] introduced the “Material Cloning” framework and directly acquired elastic parameters from images. Although estimating parameters from images has the advantage of non-invasiveness, it is difficult to assess its accuracy due to the lack of real data.

Currently, the finite-element method (FEM) is the most universally used numerical computation method in the engineering analysis field. To solve a problem, FEM subdivides a large problem into smaller, simpler parts called finite elements. These finite elements are described as a series of simple equations, which are then assembled into a larger system to model the entire problem. (https://en.wikipedia.org/wiki/Finite_element_method) FEM has been proven to be a powerful method to accurately simulate the physical and mechanical properties of elastic objects [19]. For example, Fu et al. [2] proposed a novel material reconstruction method based on FEM for the elasticity imaging of human lower legs. In addition, the study of Varga et al. [20] demonstrated that quasistatic, homogenized finite element analysis could be used to predict the mechanical properties of the proximal femora in the dynamic sideways fall situation.

Compared with other modeling methods, FEM possesses higher accuracy. However, soft tissues were considered as linear materials in the earlier studies. For example, Chikayoshi et al. [21] treated soft tissues as linear, elastic, incompressible material by assuming a constant shear modulus at the boundary of the region of interest. Mcgrath et al. [22] used an iterative method to update Young's modulus of soft tissues based on numerically calculated stress distributions, assuming the material is linearly elastic. Bickel et al. [8] acquired a set of example deformations of real objects (a pillow, a foam block, and human face) and modeled these materials by non-linear interpolation of their stress–strain relationships in strain-space. Although this modeling technique [8] was referred to be non-linear, their experiments focused on the visual evaluation of the surface deformations and lacked the quantitative evaluation.

To perform parameter estimation, optimization algorithms are usually used to calculate the material parameters [2], [23], [24]. During the optimization procedure, the initial values of the parameters to be solved may affect the astringency and outputs of the optimization algorithm. Consequently, three challenges for the parameter estimation should be addressed. These include: (1) how to obtain accurate stress and strain values of soft tissues; (2) how to build a model for solving the nonlinear parameters of soft tissues; and (3) how to design an optimization algorithm and set suitable initial values to gain highly precise optimization algorithm results. To address these challenges, we propose a novel parameter estimation method combining FEM and an improved Levenberg–Marquardt (LM) algorithm based on the data obtained using an in-house data acquisition platform.

## Method

**Figure 1:**
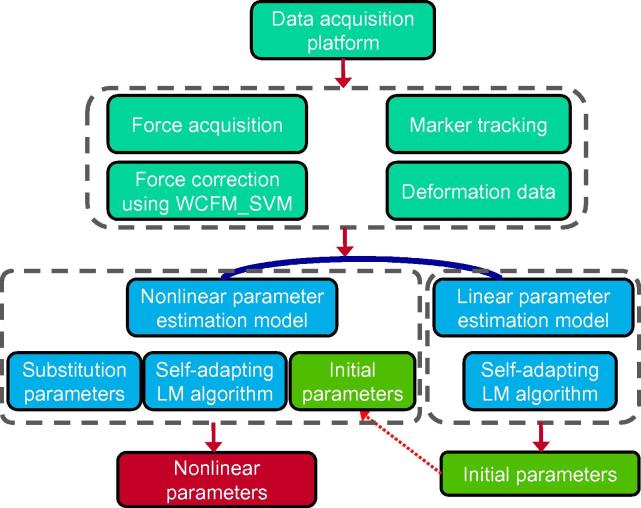
**Schematic illustration of the proposed nonlinear parameter estimation method** We firstly acquired forces and their corresponding deformation data of the experimental material using our data acquisition platform. The measured forces were corrected using the presented WCFM_SVM model. A nonlinear parameter estimation model was then established based on FEM, which introduced substitution parameters to avoid solving complicated nonlinear problems. The nonlinear parameters were solved using the presented self-adapting LM algorithm. WCFM, weighted combination forecasting model; SVM, support vector machine; FEM, finite element model; LM, the Levenberg–Marquardt.

In this section, we describe our nonlinear parameter estimation method of soft tissues. Figure 1 depicts the overview of our method. Firstly, we acquire forces and their corresponding deformation data of the experimental material using our data acquisition platform. The measured forces were corrected using the presented weighted combination forecasting model based on a support vector machine (WCFM_SVM) model so as to assure high precision. Secondly, a nonlinear parameter estimation model was established based on FEM. Substitution parameters were introduced into the model to avoid solving complicated nonlinear problems. Thirdly, the nonlinear parameters are solved using the presented self-adapting Levenberg–Marquardt (LM) algorithm. Initial parameters solved by a linear finite element model were introduced into the process of nonlinear parameter estimation.

### Data acquisition and force correction using a WCFM_SVM model

**Figure 2:**
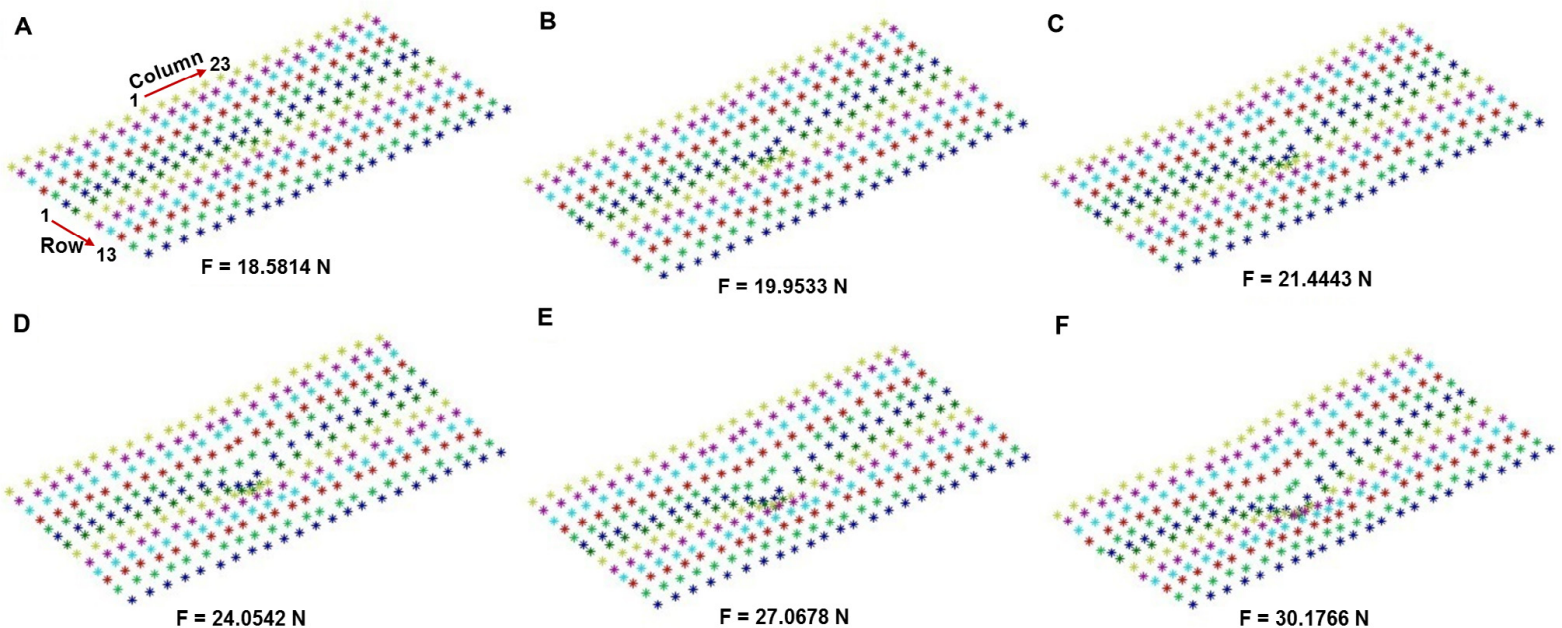
**Surface data of the experimental material acquired using PPT2** External force at six different intensities (increased from **A** to **F**) was exerted on the material. The size of the material is 50 × 30×10 cm^3^ and data were collected in a 23 × 13 matrix (23 columns and 13 rows). The force was exerted at the 150th point (the 7th row and 12th column). Each of the 13 rows on the surface of the experimental material is coded using a different color. PPT2, precision position tracker with 2 cameras; F, force; N, Newton.

In order to accurately depict the real properties of soft tissues, we exerted external forces vertically on the soft tissue surface and acquired its deformations correspondingly. The data acquisition platform consists of an optimal movement tracking device (precision position tracker with 2 cameras; PPT2) and a pressure acquisition device [25]. PPT2 can track marked targets in a 10 m × 10 m area with 1 mm precision in real time, which is used to obtain the deformation data of the soft tissue surface. The pressure acquisition device is used to obtain forces exerted on the experimental material. Given substitute materials are usually adopted as the test material in the studies on soft tissues [8], [26], a memory pillow is used as experimental material in this study, which is made of slow rebound material (polyether polyurethane) and shows dimensional stability and fold resistance. The device consists of a SDI-2F miniature pressure sensor and an Advanced RISC Machines (ARM)-based application board. The experimental material, with a size of 50 (L) × 30 (W) × 10 (H) cm^3^, was sampled evenly and 299 data points (13 rows and 23 columns) were acquired and PPT2 was used to measure the 3D coordinate information. Figure 2 shows the surface deformation obtained for the experimental material with its bottom fixed and the external forces are exerted on the 150th sampling point (the 7th row and 12th column). Markers in different lines on the material surface are color coded in each deformation. We can see that deformations of the experimental material increased gradually as the external forces increased, and the intensities of the six external forces are shown in Figure 2.

For the pressure acquisition device, the input of its force sensor is a force and the output is the corresponding voltage value. In theory, the output has a linear relation with the input. However, the acquired forces do not show a linear variability because the precision of the sensor is affected by the external environment, such as temperature and electromagnetic interference (EMI). Therefore, we present a forecasting model to correct the acquired forces. A set of samples were acquired, and each sample consisted of six data points that include the actual force and five corresponding voltages measured by the force sensor. The actual forces, which range from 0 to 3000 g, are obtained by an electronic scale with 2 g precision. A WCFM_SVM model was built from the collected samples, using the predicted value of single forecasting models as its input and the actual forces as the output. For each single forecasting model, the voltage measured by the force sensor is used as the input and the actual force as the corresponding output.

Assume that the WCFM_SVM model is constructed with m single forecasting models and n samples, the predictive value of the *j*th sample obtained from the *i*th single forecasting model is $X_{\mathrm{ij}}(i=1\text{,}2\text{,}\cdots \text{,}m\text{;}j=1\text{,}2\text{,}\cdots \text{,}n)$, and the corresponding actual value of the *j*th sample is $Y_{j}(j=1\text{,}2\text{,}\cdots \text{,}n)$. The weight of each single forecasting model in CFM is $\omega_{i}(i=1\text{,}2\text{,}\cdots \text{,}m)$, where $\omega_{i}$ varies for different single forecasting model in the WCFM_SVM model. Here, $\omega_{i}$ is calculated according to the sum of the relative error of each single forecasting model. Assume that the sum of the relative error of the *i*th single forecasting model is $E_{i}$ for n samples, the weight $\omega_{i}$ is:

(1) $$\omega_{i}=\frac{1/E_{i}}{\sum_{k=1}^{m}1/E_{k}}\text{.}$$

The weights of m single forecasting models can be calculated according to Equation (1). Next, the predicted forces of each single forecasting model and the corresponding weight were used as the input of WCFM_SVM, and the corresponding actual forces measured by the electronic scale as the output. After normalizing the aforementioned training data and updating weights of WCFM_SVM model, a force correction model was obtained. Ultimately, the trained WCFM_SVM model was used to rectify the collected data so as to obtain more precise forces.

### Modeling of nonlinear parameter estimation using FEM

The parameter estimation model was inspired by the idea of partial linearity of nonlinear materials [8]. Firstly, the acquired surface data were discretized and then the relationship between stress and strain was built by utilizing Young’s modulus and Poisson’s ratio, respectively, to describe the nonlinearity of soft tissues. Substitution parameters were then applied to calculate the unit stiffness matrix in order to avoid solving complicated nonlinear problems.

#### Discretization and stress–strain relationship

The scattered data of soft tissue deformation obtained using the data acquisition platform were numbered and divided into hexahedral finite units, each of which was further divided into five tetrahedral finite units. Parameters Young’s modulus and Poisson’s ratio varied for different hexahedral units, whereas the tetrahedrons in one hexahedron share the same parameters. Accordingly, the stress–strain relationship can be modeled using linear FEM for each tetrahedral unit.

The relationship between stress ***σ*** and strain ***ε*** is defined as ***σ*** = ***Dε***, where ***D*** is a 6 × 6 stress–strain relationship matrix, whereas ***σ*** and ***ε*** are denoted as 6 × 1 matrices. ***D*** can be described using Young’s modulus *E* and Poisson’s ratio *v*:

(2) $$D=\frac{E}{\left(1+v)(1-2v\right)}(G+vH)\text{,}$$

where Young’s modulus E defines material elasticity and Poisson’s ratio v is unitless and describes material compressibility, with ***G*** and ***H*** as two constant matrices [27]. Given that elastic matrix ***D*** is positive, definite, and the elastic material is isotropous, the Poisson’s ratio v should satisfy 0<v<0.5.

#### Nonlinear parameter estimation model

For the isotropic linear tetrahedral FEM, the element stiffness matrix $K^{e}$ describes the relationship between nodal forces and displacements, which is defined as:

(3) $$K^{e}=\int_{V^{e}}{\left(B^{e}\right)}^{T}{\mathrm{DB}}^{e}=V^{e}{\left(B^{e}\right)}^{T}{\mathrm{DB}}^{e}\text{,}$$

where $V^{e}$ is the volume of the tetrahedral element, and $B^{e}$ is the geometric function matrix. According to Equation (2), $K^{e}$ has a nonlinear relationship with E and v. In this study, substitution parameters were utilized to describe $K^{e}$. Firstly, we denote substitution parameters α=E/(1+v)(1-2v) for Equation (2), and thus D=α(G+vH)=αG+αvH. Equation (3) can be rewritten as:

(4) $$K^{e}=\alpha^{e}V^{e}{\left(B^{e}\right)}^{T}{\mathrm{GB}}^{e}+v^{e}V^{e}{\left(B^{e}\right)}^{T}HB^{e}\text{.}$$

In Equation (4), $K^{e}$ shows a weaker nonlinear relationship with $\alpha^{e}$ and $v^{e}$. After $\alpha^{e}$ and $v^{e}$ are calculated, $E_{e}$ can be obtained according to α=E/(1+v)(1-2v).

The complete stiffness matrix K(α,v) can be obtained by assembling all the element stiffness matrices. α and v are the nonlinear parameters of soft tissues. When external force F is exerted on the finite element nodal point *m* of soft tissues perpendicularly, the force can be calculated after acquiring the displacements U of the soft tissue surface using the static FEM equation K(α,v)U=F. Forces are exerted on soft tissues perpendicularly, and the calculated external forces $f{}_{i}$ should be close to the measured forces ${\overset{∼}{f}}_{i}$ for each finite element node, allowing for estimation of α and v by solving a minimization problem:

(5) (α^,v^)=argmin(α,v)∑i=1n‖fi(α,v)-f∼i‖2,

where $f{}_{i}(\alpha \text{,}v)=K_{i}(\alpha \text{,}v)u_{i}$, ${\overset{∼}{f}}_{i}$, and $u_{i}$ are the measured external forces and the corresponding displacements, respectively.

Given the potential local minima resulting from the minimization problem, initial parameters of soft tissues were introduced to improve the Equation (5) as follows:

(6) (α^,v^)=argmin(α,v)∑i=1n‖fi(α,v)-f∼i‖2+γ∑j=1k‖αj-α^0‖2+‖vj-v^0‖2,

where α^0 and v^0 are the initial parameters of soft tissues, and γ is used to adjust the influence of parameters toward optimal results.

### Parameter estimation algorithm

#### Self-adapting Levenberg–Marquardt algorithm

The LM algorithm is usually used to solve the optimization problem minS(x), where $S(x)=Y{\left(x\right)}^{T}\;\text{Y}(x)=\sum_{i=1}^{m}Y_{i}^{2}(x)$. The algorithm is a trust-region method, and the values of the target function are required to descend in each iteration step. The search direction of the current iteration point for the traditional LM algorithm [1] is:

(7) $$d_{k}(\lambda_{k})=-{\left(J_{k}^{T}J_{k}+\lambda_{k}I\right)}^{-1}J_{k}^{T}Y{}_{k}\text{,}$$

where $J_{k}$ is the Jacobian matrix. To prevent $J_{k}^{T}J_{k}$ from becoming too big when it approaches singularity during the iteration process $\lambda_{k}$ was introduced as a positive parameter. The choice of parameter $\lambda_{k}$ is essential to the LM algorithm. In this study, $\lambda_{k}$ is defined as:

(8) $$\lambda_{k}=\eta_{k}\left(\theta \frac{\min\left(\|Y{}_{k}{\|}_{2}^{2}\text{,}\|J_{k}^{T}Y{}_{k}{\|}_{2}^{2}\right)}{\left\|Y{}_{k}\right\|}+(1-\theta )\frac{\min\left(\|Y{}_{k}{\|}_{2}^{2}\text{,}\|J_{k}^{T}Y{}_{k}{\|}_{2}^{2}\right)}{\left\|J_{k}^{T}Y{}_{k}\right\|}\right)\text{,}$$

where θ∈[0,1], $\eta_{k}$ is the self-adaptive factor of parameter $\lambda_{k}$, and the relationship of $\eta_{k}$ between neighboring iteration steps is:

(9) $$\eta_{k+1}=\min\{\text{c,}\eta_{k}q(\tau_{k}))\text{,}$$

where c is a positive constant, which is determined by the actual optimization problems. q(τ) is a nonnegative continuous function of τ .

(10) $$q(\tau )=\max\{0.5\text{,}1-{\left(2\tau -1\right)}^{2}\}\text{.}$$

The introduction of q(τ) was used to adaptively adjust $\lambda_{k}$. τ is a criterion that testifies the validity of the iteration step and is used to decide acceptance or rejection. The definition of τ is denoted as:

(11) $$\tau_{k}=\frac{\|Y{}_{k}{\|}_{2}^{2}-\|Y(x_{k}+d_{k}){\|}_{2}^{2}}{\|Y{}_{k}{\|}_{2}^{2}-\|Y{}_{k}+J_{k}d_{k}){\|}_{2}^{2}}\text{,}$$

where $\tau_{k}$ defines the ratio of the actual decrement to the forecasting decrement during the iteration process. When $\tau_{k}$ is bigger than the given threshold, the iteration step $d_{k}$ is accepted and the self-adapting factor $\eta_{k}$ is adjusted. Otherwise, the step is rejected. That is:

(12) $$x_{k+1}=\left\{\begin{array}{c} x_{k}+d_{k}\text{,}\tau_{k}>p_{0}\\ x_{k}\text{,}\;\mathrm{others} \end{array}\right)\text{.}$$

The self-adapting LM algorithm is described in Algorithm 1

**Table:** 

**Algorithm 1** Self-adapting LM algorithm
1: provide the initial parameters $x_{1}\in R^{n}\text{,}$ initialize constant ε,θ∈[0,1], *c* > 0, 0 < *p*_0_ < 1, *η*_1_ > *c* 2: **while** $\|J_{k}^{T}Y{}_{k}{\|}_{2}⩾\varepsilon$, **do** 3: calculate iterative step $d_{k}$ according to Equations (7), (8) 4: calculate trade-offs indicator $\tau_{k}$ according to Equation (11) 5: **if** $\tau_{k}>p_{0}$ **then** 6: $x_{k+1}\leftarrow x_{k}+d_{k}$ 7: **else** 8: $x_{k+1}\leftarrow x_{k}$ 9: **end if** 10: calculate adaptive factor $\eta_{k+1}$ according to Equations (9), (10) 11: **end while**

#### Solving of initial parameters

The initial parameter $x_{1}$ is of great importance to the self-adapting LM algorithm proposed in this study. In addition, the initial parameters were introduced into our nonlinear parameter estimation model.

Although Young’s modulus and Poisson’s ratio in different parts of soft tissues are different, the value of the parameters should fluctuate around a certain numerical value, which can be considered as the average parameter. Nonlinear soft tissue can be regarded as a linear material [21], [22]. Therefore, a linear model can be built to calculate the equivalent parameters for linear soft tissue, which are used as the initial parameters in the nonlinear parameter estimation model and the initial values in the self-adapting LM algorithm.

Denote that ϕ(v)=1/(1+v)(1-2v) and φ(v)=v/(1+v)(1-2v), and thus D=E(ϕ(v)G+φ(v)H) according to Equation (2). Element stiffness matrix $K^{e}$ can be rewritten as:

(13) $$K^{e}(E\text{,}v)=V^{e}E{\left(B^{e}\right)}^{T}(\phi (v)G+\varphi (v)H)B^{e}\text{.}$$

The initial parameters E^0 and v^0 can be obtained by solving the following minimization problem (please see File S1 for detail):

(14) (E^0,v^0)=argmin(E,v)∑i=1n‖ENi(v)-f∼i‖2.

The initial substitute parameter α^0 can be calculated according to α=E/(1+v)(1-2v), and then the initial substitution parameters (α^0,v^0) can be used for the initial parameter estimation model and the initial value $x_{1}$.

## Results and discussion

Currently, there are no public data available for evaluating the performance of parameter estimation methods for soft tissues. In this study, a memory pillow was used as the experimental material.

We quantitatively evaluated the performance of the parameter estimation method by comparing the measured nodal displacements and the nodal displacements calculated using the parameters obtained. To do so, we first exerted the external force of six different intensities on the material (Figure 2, A–F), followed by the acquisition of their values and the corresponding deformation data using an in-house data acquisition platform. Next, WCFM_SVM force correction model was adopted to correct the measured forces that were used to verify the effectiveness of our nonlinear parameter estimation model.

### Comparison of force correction using WCFM_SVM model and other forecasting models

To validate the accuracy of the WCFM_SVM force correction model, 76 sets of samples, including forces acquired using our pressure acquisition device and their actual forces acquired using an electronic scale, were collected and divided into a training set (70 sets of samples) and a test set (6 sets of samples). The performance of the WCFM_SVM model was compared with that of seven other forecasting models. These include a polynomial fitting method, three single forecasting models back propagation (BP) neural network, SVM, and least squares support vector machine (LSSVM)), a CFM based on SVM (CFM_SVM), a CFM based on LSSVM (CFM_LSSVM), and a WCFM based on LSSVM (WCFM_LSSVM). The weights of the single forecasting models for CFM_SVM and CFM_LSSVM were the same. The corrected forces obtained using the eight correction models were compared with the actual forces acquired using an electronic scale.

**Figure 3:**
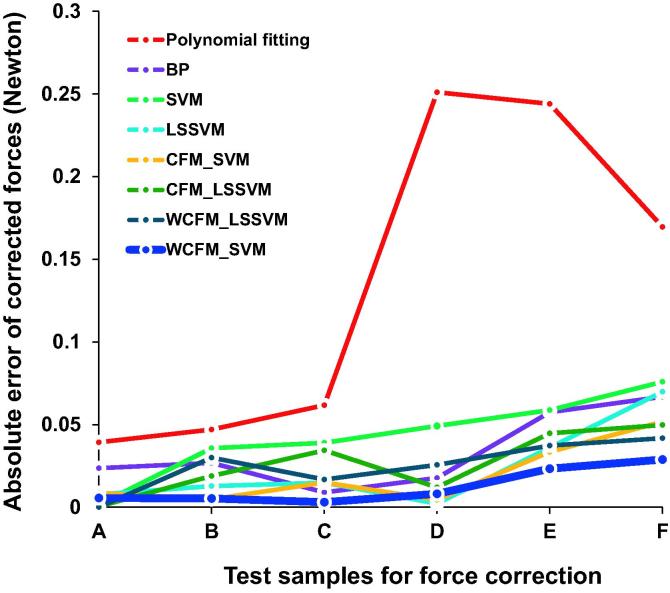
**Absolute error using different force correction models** External forces at six different intensities (increased from A to F) were exerted on the material. The absolute errors between the actual forces acquired using the electrical scale and the correct forced obtained using different correction models were plotted. Absolute errors of our proposed WCFM_SVM model are smaller across different sample groups even if relatively large forces are applied, showing the strong robustness of our proposed WCFM_SVM model. BP, back propagation; SVM, support vector machine; LSSVM, least squares SVM; CFM, combination forecasting model; WCFM, weighted CFM.

The absolute errors between the corrected forces and the actual forces for samples A–F are shown in Figure 3. Absolute errors tended to increase when external forces were applied with higher intensities. Compared to other models, absolute errors for the proposed WCFM_SVM model remained relatively low. The max absolute error of the proposed WCFM_SVM model is less than 0.03 Newton (N), which is smaller than those obtained using other models. These data indicate that compared to other correction models, the proposed WCFM_SVM model presented the smaller absolute error and showed stronger robustness.

The mean absolute error ($e_{\text{MAE}}$), square error ($e_{\text{MSE}}$), and absolute percent error ($e_{\text{MAPE}}$) between the actual and corrected forces are shown in Table 1. Mean absolute error is calculated as eMAE=1/n∑i=1n|xi-x^i|, whereas mean square error is calculated as eMSE=1/n∑i=1n(xi-x^i)2, and mean absolute percent error is calculated as eMAPE=1/n∑i=1n|(x^i-xi)/xi|×100%. In this study, $x_{i}$ denotes actual forces, x^i denotes corrected forces, and *n* indicates the number of samples (n=6). Compared with the other forecasting models, our WCFM_SVM correction model shows a better performance thus can provide accurate forces for the parameter estimation model.

**Table 1:** **Mean errors of forces generated using different force correction models**

**Model**	**Mean absolute error (Newton)**	**Mean square error (Newton)**	**Mean absolute percent error (%)**
Polymodal fitting	0.1286	2.6559	1.3387
BP	0.0323	0.2276	0.2837
SVM	0.0326	0.1515	0.2745
LSSVM	0.0312	0.2399	0.1975
CFM_SVM	0.0215	0.1120	0.1399
CSM_LSSVM	0.0325	0.1640	0.2240
WCFM_LSSVM	0.0312	0.0146	0.2291
**WCFM_SVM**	**0.0195**	**0.0830**	**0.1333**

*Note*: The model proposed in this study is put in bold. BP, back propagation; SVM, support vector machine; LSSVM, least squares SVM; CFM, combination forecasting model; WCFM, weighted CFM.

### Parameter estimation using linear FEM

Many earlier studies have considered soft tissues as linear materials [21], [22]. Accordingly, we first considered the experimental material as a linear object to calculate initial parameters E^0 and v^0 for the experimental material across six different deformations. As shown in Table 2, the initial parameters E^0 and v^0 varied for different deformations, indicating that the experimental material tested is nonlinear.

**Table 2:** **Initial parameters solved using a linear FEM**

**Deformation set**	**Young’s modulus (Pascal)**	**Poisson’s ratio**	**Corrected forces by WCFM_SVM (Newton)**
A	7.4065 × 10^3^	0.07	18.5814
B	7.9424 × 10^3^	0.06	19.9533
C	6.2843 × 10^3^	0.06	21.4443
D	7.2075 × 10^3^	0.06	24.0542
E	7.3448 × 10^3^	0.07	27.0678
F	8.4128 × 10^3^	0.06	30.1766

*Note*: The deformation sets are named according to the panel indexes in Figure 1. FEM, finite element model.

**Figure 4:**
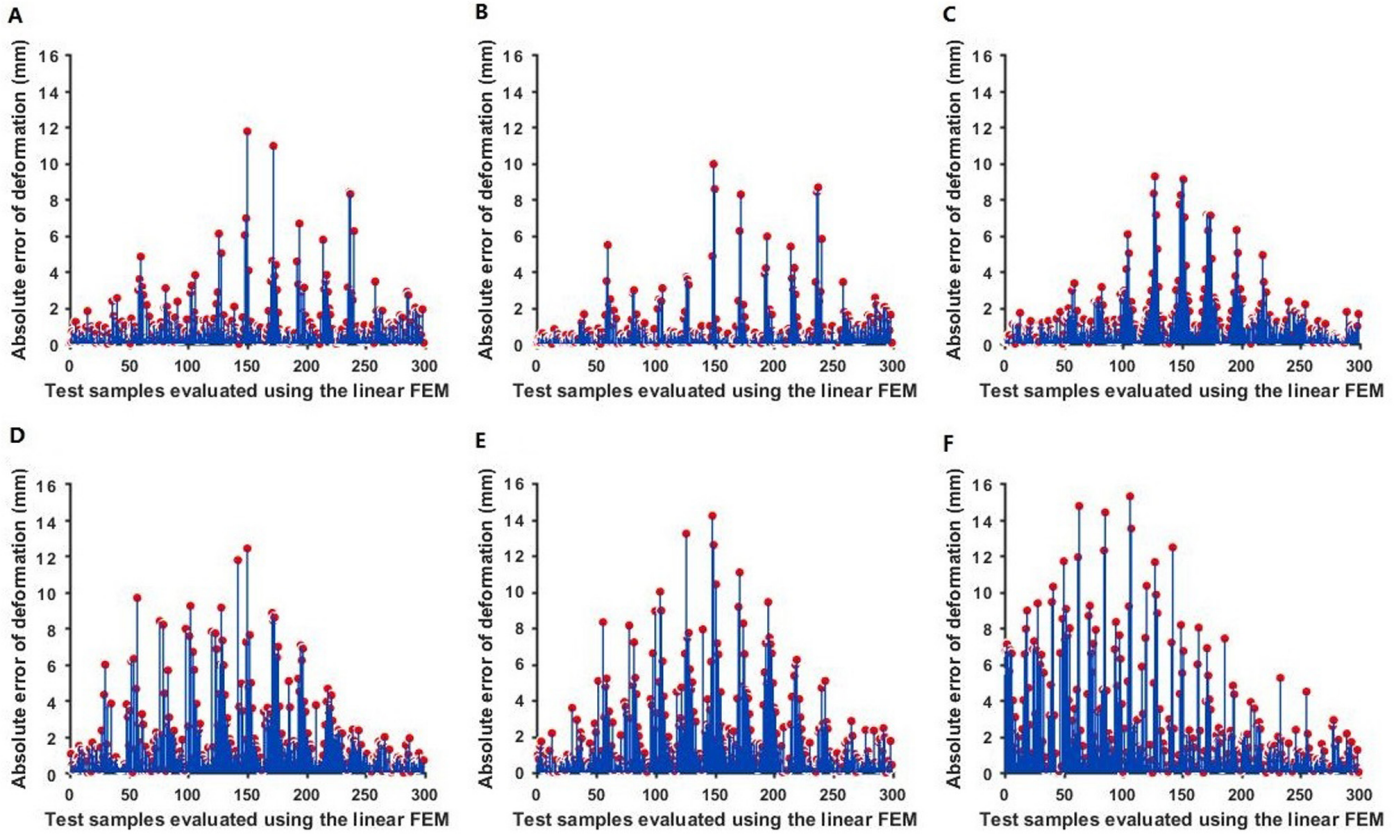
**Absolute errors between the measured nodal displacements and the calculated nodal displacements using the linear FEM** External forces at six different intensities (increased from **A** to **F**) was exerted on the material. Deformation was sampled evenly in a 23 × 13 matrix for the surface with the size of 50 × 30 cm^2^. The force was exerted at the 150th point (the 7th row and 12th column). The red dots denote the values of the absolute error between the nodal displacements measured using PPT2 and nodal displacements calculated using linear FEM.

The initial parameters shown in Table 2 were input into the linear FEM to calculate nodal displacements. The calculated and measured nodal displacements were compared. As shown in Figure 4, there were large absolute errors between the calculated nodal displacements and the measured displacements, especially for larger deformations. Therefore, if a nonlinear material is regarded as a linear object, the estimated elastic properties using linear model are likely to possess considerable error.

### Parameter estimation using the proposed nonlinear model

**Figure 5:**
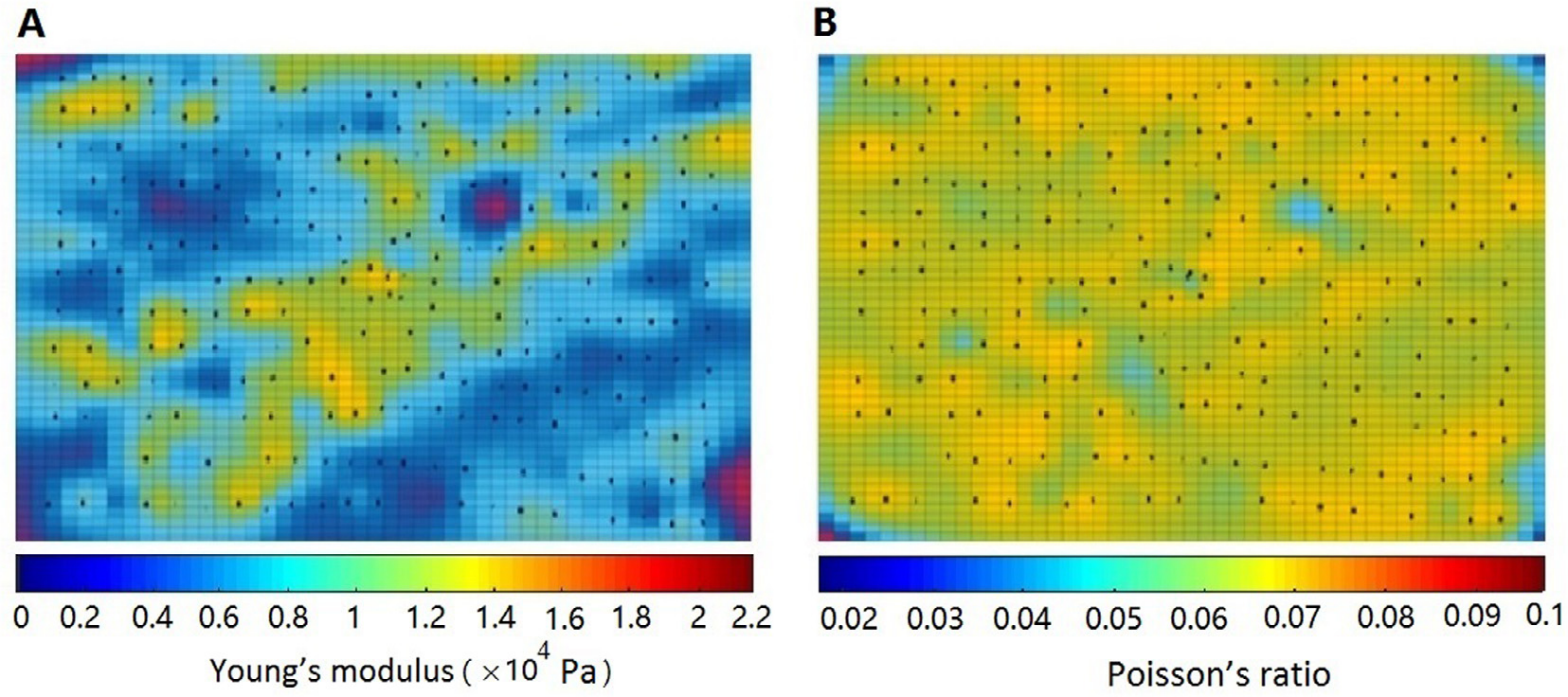
**Distribution of Young’s modulus and Poisson’s ratio** Young’s modulus E (**A**) and Poisson’s ratio v (**B**) for the material were calculated using the self-adapting LM algorithm. Neighboring hexahedral elements shared similar colors, showing that parameters of neighboring hexahedral elements are similar to each other in local regions. Pa, Pascal.

We next applied the proposed parameter estimation method to describe the nonlinearity of the experimental material, and build the relationship between the parameters to be solved, *i.e.*, the deformations and the external forces. The model proposed was based on the partial linearization of nonlinear material. Although five tetrahedral elements appear to be linear in one hexahedral element, the overall nonlinear relationship between stress and strain enables the calculation of the nonlinear parameters of the experimental material. Young’s modulus E and Poisson’s ratio v of the experimental material were obtained using the self-adapting LM algorithm. As shown in Figure 5, Young’s modulus and Poisson’s ratio of neighboring hexahedral elements are rather close to each other in local regions, thereby appearing to be linear in local areas. However, the material presents nonlinearity from the perspective of the entirety. These observations demonstrate the partial linearization of nonlinear material, providing the theoretical basis for the proposed parameter estimation method of soft tissues.

Bickel et al. [8] proposed a modeling technique for simulating non-linear heterogeneous soft tissues. However, this study lacks quantitative evaluation. In order to further quantitatively verify the effectiveness of our nonlinear parameter estimation model, each group of 299 scattered deformation data was divided into a sample set (249 data points) and a test set (50 data points). The sample set was utilized to solve the parameters of the experimental material, which were used to calculate the nodal displacements of the test set. The absolute errors in nodal displacements were obtained by comparing the calculated nodal displacements with the measured nodal displacements of the test set.

**Figure 6:**
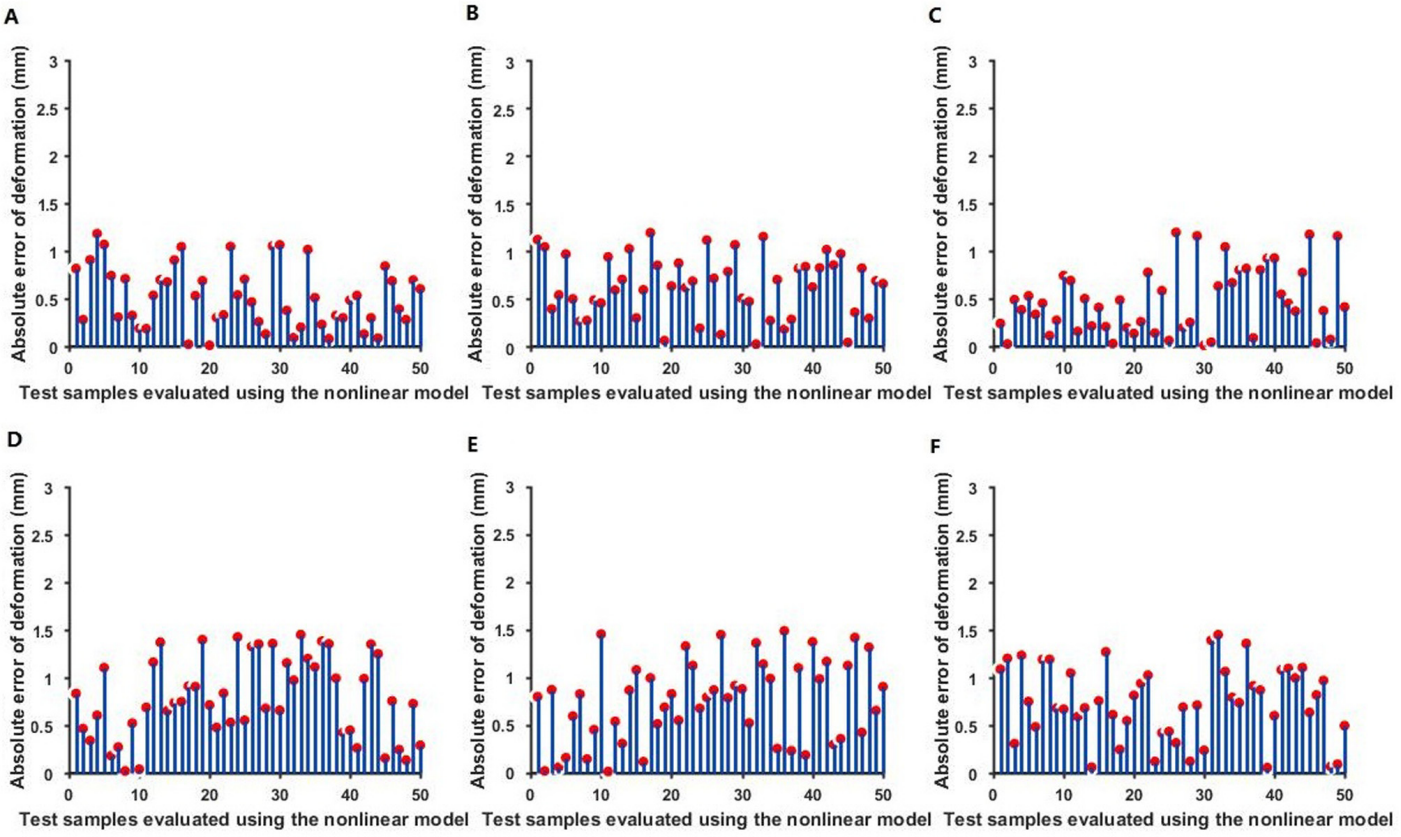
**The absolute error between the measured nodal displacements and the nodal displacements calculated using the proposed nonlinear method** External forces at six different intensities (increased from **A** to **F**) was exerted on the material. Deformation data at each external force were divided into a sample set (249 data points) and a test set (50 data points). Parameters solved from the sample set were used to calculate the nodal displacements of the test set. The red dots denote the values of the absolute error between the calculated nodal displacements and measured nodal displacements.

As shown in Figure 6, the absolute errors in nodal displacement were low. However, when regarding the nonlinear material as a linear object [21], [22] for parameter solving, the nodal displacement errors were very large (Figure 4, Figure 6). Therefore, the proposed nonlinear method performs better than linear FEM with small errors.

Table 3 shows the maximum error $d_{\text{max}}$ and the average error $d_{\text{mean}}$ of nodal displacements calculated using the linear FEM and the proposed nonlinear method respectively. The maximum error of the proposed nonlinear method was less than 1.5 mm, far less than that using the linear FEM, which was around 10 mm. Therefore, the parameters estimated using our method are more accurate than those estimated using the linear FEM.

**Table 3:** **Difference in the nodal displacement calculated using the linear FEM and the proposed nonlinear model**

**Deformation set**	**Linear FEM (mm)**		**The proposed nonlinear model (mm)**	
	**Maximum error**	**Average error**	**Maximum error**	**Average error**
A	11.75	1.22	1.18	0.52
B	9.94	0.98	1.19	0.62
C	9.26	1.45	1.20	0.47
D	12.4	2.11	1.45	0.79
E	14.2	2.30	1.50	0.76
F	15.3	2.80	1.44	0.74

## Conclusion

In this study, a novel nonlinear parameter estimation method was proposed for soft tissues. We collected deformation data of a memory pillow to mimic soft tissues using the in-house data acquisition platform. To provide precise data for the parameter estimation model, a WCFM_SVM model was constructed to correct measured forces. A tetrahedral finite element model was constructed based on the concept of partial linearization of the nonlinear material. The model utilized substitution parameters of Young’s modulus and Poisson’s ratio to describe the physical characteristics of soft tissues rather than directly calculating them, thereby avoiding solving complicated nonlinear problems. In addition, we introduce the initial parameters of soft tissues into our parameter estimation model to improve model robustness and obtain more accurate parameters. The presented self-adapting LM algorithm was utilized to solve the nonlinear parameter estimation model while a linear finite element model provided the initial values for the optimization algorithm.

We further quantitatively analyze the error between the calculated and measured nodal displacements by comparing the performance of the proposed method with that of the linear finite model. Our results indicate that the proposed parameter estimation method is of high accuracy. PPT2 was applied to locate the position of the circular markers on the memory pillow during the measurement. However, the volume of the circular markers may introduce deviations in nodal displacement. In future studies, we will adopt other more precise methods to obtain the surface data to build a parameter estimation model of higher precision. In addition, as only the parameters that describe the physical characteristics of soft tissues were obtained, a dynamic deformation process for soft tissues will be simulated, adapting the parameters estimated using the proposed parameter estimation method in such future work.

## Authors’ contributions

QT and ZY conceived the idea and supervised the study. XL measured the deformations and corresponding external forces using the data acquisition platform. QT and MZ designed and implemented the proposed method, and analyzed the results. QT drafted the manuscript. WZ and GZ edited the manuscript. All authors read and approved the final manuscript.

## Competing interests

The authors have declared no competing interests.

## Supplementary material

File S1 Solving initial parameters.
