# Supplementary material for: A Novel Nonlinear Parameter Estimation Method of Soft Tissues
Source: Genomics Proteomics Bioinformatics. 2017 Dec 13;15(6):371–80. doi: 10.1016/j.gpb.2017.09.003 (PMC5828669; doi:10.1016/j.gpb.2017.09.003)
Supplement: File S1 — Solving initial parameters. [file mmc1.docx]

**File S1 Solving initial parameters**

The initial parameter $\mathbf{x}_{1}$ is of great importance to the self-adapting LM algorithm proposed in this study. In addition, the initial parameters were introduced into our nonlinear parameter estimation model. A linear model was built to calculate$\mathbf{x}_{1}$.

As described in the main text, the relationship between stress $\mathbf{\sigma}$ and strain $\mathbf{\varepsilon}$ is defined as $\mathbf{\sigma}=\mathbf{D\varepsilon}$, where$\mathbf{D}$ is a $6\times6$stress−strain relationship matrix, whereas $\mathbf{\sigma}$ and $\mathbf{\varepsilon}$ are denoted as $6\times1$matrices. $\mathbf{D}$ can be described using Young’s modulus $E$ and Poisson’s ratio $v$:

$\mathbf{D}=\frac{E}{(1+v)(1-2v)}(\mathbf{G}+v\mathbf{H})$. （1）

Firstly, Equation 1 can be rewritten as:

, （2）

where and are denoted as follows:

, （3）

. （4）

Hence, the element stiffness matrix $\mathbf{K}^{e}$ is denoted as:

. （5）

The relationship between the complete stiffness matrix , displacements $\mathbf{U}$, and external forces $\mathbf{F}$ is . Assuming that $\mathbf{N}(v)$ is:

, （6）

thus $E\mathbf{N}(v)=\mathbf{F}$. For all $n$ nodes, the initial parameters $\widehat{E}_{0}$ and $\widehat{v}_{0}$ are:

$(\widehat{E}_{0},\widehat{v}_{0})=\mathop{\mathrm{argmin}}\limits_{(E,v)} \left\{\sum_{i=1}^{n} \left\|E\mathbf{N}_{i}(v)-\widetilde{\mathbf{f}}_{i}\right\|^{2}\right\}$. （7）

In this study, forces are exerted on the nodal point $m$(the 150th point, that is the 7th row and 12th column), and the external forces of all other moving nodes are zero. Therefore, the initial Poisson’s ratio $\widehat{v}_{0}$ is

$\widehat{v}_{0}=\mathop{\mathrm{argmin}}\limits_{v} \left\{\sum_{i=1,i\neq m}^{n} \left\|\mathbf{N}_{i}(v)-\mathbf{0}\right\|^{2}\right\}$, （8）

thus, the initial Young’s modulus $\widehat{E}_{0}$ is

$\widehat{E}_{0}=f_{m}/N_{m}(\widehat{v})$. （9）

The initial substitute parameter $\widehat{\alpha}_{0}$ can be obtained according to $\alpha=E/(1+v)(1-2v)$, and then the initial substitution parameters $(\widehat{\alpha}_{0},\widehat{v}_{0})$ can be used for the initial parameters of the nonlinear parameter estimation model and the initial value $\mathbf{x}_{1}$.
